# Clinical ocular prediction model of postoperative ametropic amblyopia in patients with congenital ectopia lentis

**DOI:** 10.3389/fmed.2024.1491736

**Published:** 2024-10-21

**Authors:** Xinyue Wang, Linghao Song, Yan Liu, Qiuyi Huo, Yang Sun, Zexu Chen, Wannan Jia, Xin Shen, Yalei Wang, Xinyao Chen, Tianhui Chen, Yongxiang Jiang, Rui Wang

**Affiliations:** ^1^Department of Ophthalmology, The First Affiliated Hospital of Northwest University, Xi’an, China; ^2^Eye Institute and Department of Ophthalmology, Eye & ENT Hospital, Fudan University, Shanghai, China; ^3^Key Laboratory of Myopia and Related Eye Diseases, NHC; Key Laboratory of Myopia and Related Eye Diseases, Chinese Academy of Medical Sciences, Shanghai, China; ^4^Shanghai Key Laboratory of Visual Impairment and Restoration, Shanghai, China

**Keywords:** congenital ectopia lentis, ametropic amblyopia, nomogram, prediction model, visual prognosis

## Abstract

**Introduction:**

Despite prompt and appropriate surgical management, a considerable proportion of patients with congenital ectopia lentis (CEL) suffer from postoperative ametropic amblyopia. To predict and identify at-risk patients early, and ensure timely amblyopia treatment, we conducted a thorough investigation into the onset and progression patterns of postoperative amblyopia in patients with CEL. Moreover, an ocular prediction model was constructed for amblyopia.

**Methods:**

In this prospective cohort study, amblyopia analysis was conducted to reveal the prevalence of postoperative amblyopia at different time points of follow-up. Comparative analysis and logistic regression analysis were performed for the development of an amblyopia prediction model. Receiver Operating Characteristic (ROC) analysis, calibration plots, and decision curve analysis (DCA) were used to evaluate the performance of the model. A nomogram was created to determine the probability of postoperative amblyopia. Amblyopia was diagnosed according to the most recent edition of the Amblyopia Preferred Practice Pattern.

**Results:**

A total of 889 eyes from 677 patients operated for CEL were enrolled in this study. In the pediatric cohort, the prevalence of amblyopia showed a decreasing trend with follow-up time from 1 month to 3.5 years. A prediction model based on preoperative best-corrected visual acuity (BCVA) and cardiac phenotype was established to predict postoperative amblyopia. For effective individual prediction, a nomogram was created. With great calibration, discrimination, and clinical usefulness, the prediction model demonstrated good performance.

**Conclusion:**

The findings underscore that the prevalence of ametropic amblyopia in pediatric CEL patients who underwent lens surgery exhibited a marked decline over time. The prediction model established with preoperative BCVA and cardiac phenotype can provide accurate and individualized predictions of postoperative amblyopia, and it has the potential to assist ophthalmologists in rapidly identifying high-risk patients.

## Introduction

Congenital ectopia lentis (CEL) is a zonular dysplasia characterized by the displacement of the crystalline lens (1, 2). With an estimated incidence of 6.4 per 100,000 individuals, CEL represents the second leading cause for lens surgery in children, subsequent to congenital cataracts (3). It is frequently associated with some hereditary disorders, along with various developmental ocular abnormalities (4, 5). In severe instances, CEL may predispose patients to a series of ocular complications, including secondary glaucoma, corneal endothelial damage, and retinal detachment (3).

Current management strategies revolve around surgical treatment of ectopia lentis (6, 7). Unfortunately, despite prompt and appropriate surgical management, a considerable proportion of patients suffer from postoperative ametropic amblyopia, which is a critical determinant of visual prognosis (8–10). Amblyopia can result in substantial reductions in spatial acuity in the affected eye, compromised fine motor skills and coordination, and diminished or absent ability to perceive stereoscopic vision (11–14). These visual deficits can subsequently impair a child’s academic achievements and social interactions (15). Consequently, meticulous follow-up and strict emphasis on timely amblyopia therapy are necessary in these eyes (9).

Current studies suggest that amblyopia treatment should commence before the closure of the critical window for visual development (15, 16). The efficacy of conventional treatments, including patching, atropine, and Bangerter filters, tends to decline with advancing age, particularly beyond 7 years old (15, 16). However, there is a notable paucity of research focusing on the early identification and prediction of CEL patients at risk for postoperative amblyopia, making it extremely challenging to ensure timely intervention. Furthermore, there is a pressing need for a more nuanced understanding of the progression of postoperative amblyopia, highlighting a significant gap in existing research.

In this article, we conduct a longitudinal investigation of ametropic amblyopia among patients with CEL who underwent lens surgery at the Eye and ENT Hospital of Fudan University. Our investigation was structured around three primary objectives: first, to chart the prevalence of postoperative amblyopia across different follow-up intervals in pediatric and adults’ cohorts, respectively; second, to discern potential predictors of postoperative amblyopia by conducting a comparative analysis of preoperative demographic, ocular, cardiovascular, and genetic data between amblyopic and non-amblyopic groups; third, to develop a prediction model as well as a nomogram for the risk of preoperative amblyopia, thereby enhancing ophthalmologists’ capacity to manage amblyopia and ensuring that the optimal treatment window is not missed.

## Materials and methods

### Ethics statement

This longitudinal study enrolled subjects who underwent lens surgery from June 2017 to February 2024 at the Eye and ENT Hospital of Fudan University, Shanghai, China. The Guidelines for Transparent Reporting of a Multivariable Model for Individual Prognosis or Diagnosis (the TRIPOD statement) were followed in this study. The study was approved by the Human Research Ethics Committee of the Eye and ENT Hospital of Fudan University (ChiCTR2000039132). The study adhered to the tenets of the Declaration of Helsinki. All participants provided signed informed consent.

### Participants

**Figure 1:**
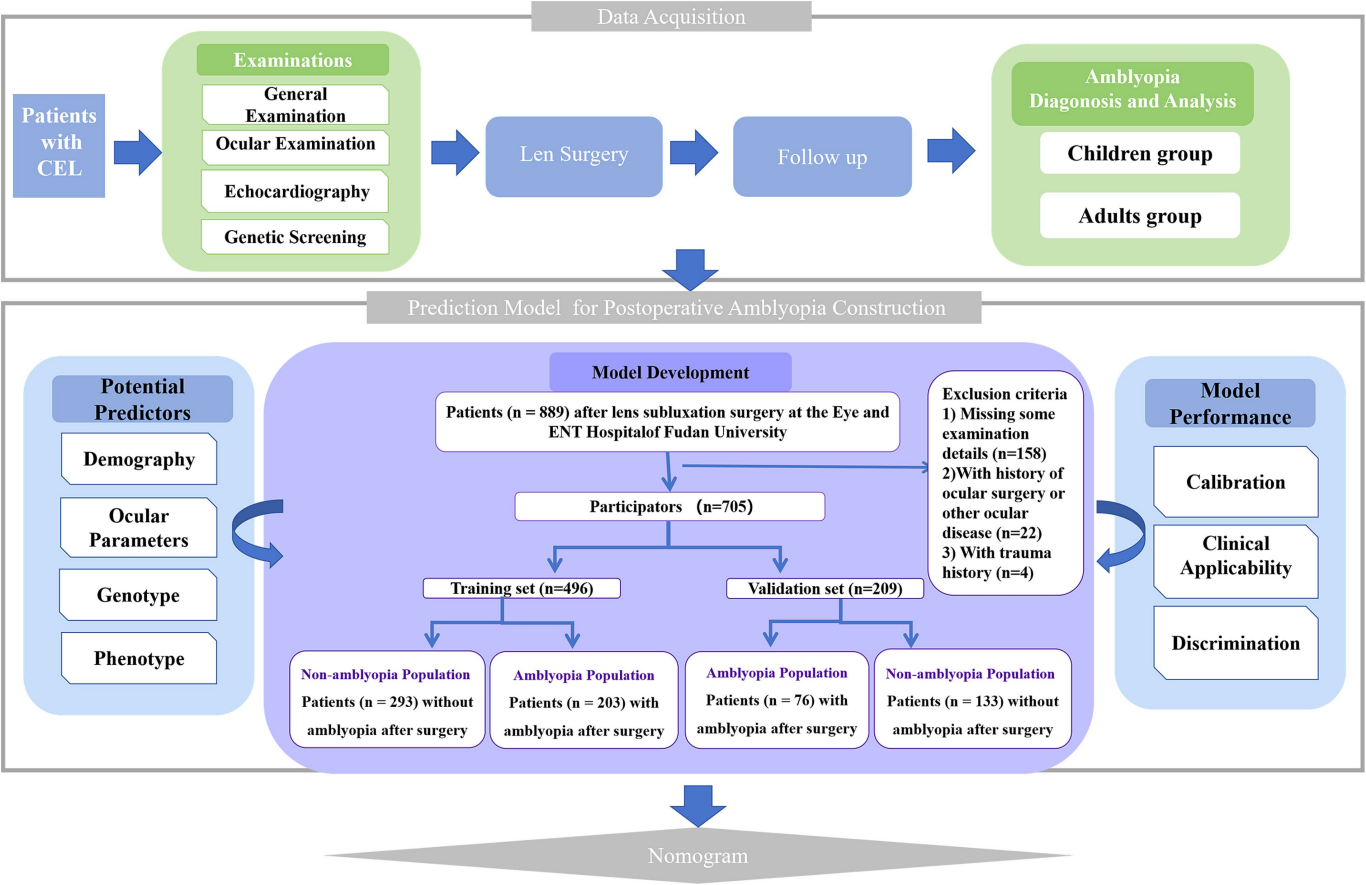
Flowchart of model development. CEL, Congenital Ectopia Lentis.

A total of 677 patients (889 eyes) with moderate EL at the Eye and ENT Hospital of Fudan University were prospectively investigated and screened for suitability for this study. Patients missing important examination details, or with microspherophakia, keratoconus, previous history of ocular surgery, uveitis, corneal disease, glaucoma, retinal detachment, or use of contact lenses in the 2 weeks prior to the examinations were excluded from this study. The flow chart of the study is shown in Figure 1. In amblyopia groups, only patients with ametropic amblyopia rather than other types of amblyopia were included. Patients with lens edge uncovered 25 to 50% of the dilated pupil were diagnosed with moderate EL (8).

### Preoperative examinations

The family and medical histories were recorded for all subjects before the examinations. All enrolled subjects underwent routine preoperative examinations and comprehensive ocular examinations. The preoperative best-corrected visual acuity (BCVA) was assessed by the same skilled optometrist. Ocular parameters including AL, corneal keratometry [flattest K (K1), steepest K (K2), mean K (Km)], horizontal corneal diameter (WTW), and anterior chamber depth (ACD) were collected by partial coherence interferometry (the IOLMaster 500, Carl Zeiss Meditec, Jena, Germany, was used from June 2017 to March 2018; the IOLMaster 700, Carl Zeiss Meditec, Jena, Germany, was used from March 2018 to February 2024). All measurements were made with a pupil scan of 6 mm in diameter. Subjects were examined by experienced ophthalmologists who were trained in the use of all devices. All values in individual subjects were taken as the means of five repeated measurements for each eye recorded by the equipment. Additionally, because AL and WTW were progressive and age-dependent, to rule out the confounding factors of age, the z-AL and z-WTW were calculated with the formula Z-score = (measured score − normative score)/normative standard deviation (SD), compared with a reference standard (17).

Genetic screening and echocardiography were performed for the patients with CEL, according to our previous study (18). Based on the echocardiography results, cardiac phenotype was categorized into normal (no cardiac manifestations), regurgitation (conditions such as mitral regurgitation or aortic regurgitation), and organic (heart with any kind of organic lesions including aortic root dilation, mitral valve elongation, mitral valve prolapse).

### Surgical procedure

All surgeries were operated by one surgeon (Dr. Y.-X. Jiang). The phacoemulsification procedure, modified capsular tension ring (MCTR) implantation or capsular tension ring and capsular hook (CTR-CH) implantation were conducted as previously described (19). In cases where a peripheral extension or tearing of the capsulorhexis rim occurred, a single-piece foldable IOL was sutured to the sclera using double-strand 9–0 polypropylene (MANI Inc., Tochigi, Utsunomiya, Japan) after anterior lensectomy combined with capsulotomy (23G; Alcon Laboratories Inc., Fort Worth, TX, United States) through a limbal approach (20).

Postoperatively, Levofloxacin Eye Drops (Santen Pharmaceuticals, Inc., Osaka, Japan) and Pred Forte Eye Drops (Allergan Pharmaceuticals, Inc., Dublin, Ireland) were applied three times daily for 1 month. Additionally, 0.1% pranoprofen (Sumika Finechem, Osaka, Japan) was applied three times daily for 1 month, followed by weekly tapering.

### Diagnosis and analysis of postoperative amblyopia

The best-corrected visual acuity (BCVA) was assessed by the skilled optometrist at every follow-up visit. Amblyopia was diagnosed according to the most recent edition of the Amblyopia Preferred Practice Pattern (21). After surgery, patients, especially those aged < 7 years old, were recommended to receive treatment for amblyopia. Follow-up visits were scheduled at intervals of 1 month, 6 months, 12 months, and then every 6 months after the surgery. Analysis was conducted separately for the right and left eyes.

### Statistical analyses

The Kolmogorov–Smirnov test was used to confirm the normal distribution of the variables. Normally distributed data were presented as mean ± standard deviation (SD), while skewed data were expressed as median (interquartile range, IQR). Categorical variables were expressed as number and proportion as appropriate. The *χ*^2^ test and Wilcoxon rank-sum test (Mann–Whitney U test) were used to compare data between the amblyopia and no-amblyopia groups in the training and validation cohorts. Univariable logistic regression analysis was used to describe the relationship between each individual predictor variable and the prediction of amblyopia. The predictor variables showing significant associations with standard outcomes (*p* < 0.05) were collected for further multivariate logistic regression analysis. Multivariate logistic regression (forward stepwise selection and exclusion criteria of type I error = 0.1 based on likelihood ratio tests) was then performed to build the risk prediction model. All predictor variables were described as odds ratios (ORs) with 95% confidence intervals (CI) and *p* values were calculated.

Calibration and discrimination were measured to assess the validity of the prediction model. To assess the calibration, we used Hosmer–Lemeshow *χ*^2^ statistics and calibration curve. To assess the discrimination, the ROC analysis was performed to calculate the area under the ROC curve (AUC) in evaluating the performance of the model. We also examined the net benefit using decision curve analysis (DCA) with regard to clinical usefulness. The training cohort was used to build the clinical ocular prediction model. Then, we verified our model in the validation cohort. *p* values of less than 0.05 for two-tailed tests were considered statistically significant. GraphPad Prism 9.5 (GraphPad Software, California, United States) and SPSS software version 26.0 (IBM Corp, Armonk, NY) were used for all statistical analyses. R software was used to generate the nomogram, ROC analysis, calibration plots, and DCA curves.

## Results

### Patient characteristics

A total of 521 patients (705 eyes) were identified as CEL and randomly divided into a training cohort and a validation cohort at a ratio of 7:3. Table 1 summarizes the baseline characteristics of the participants, including their demographic, ocular, cardiovascular, and genetic data. The training samples were obtained from 148 patients (203 eyes) with ametropic amblyopia and 195 patients (293 eyes) without amblyopia after lens surgery. The validation cohort consisted of 67 patients (76 eyes) with ametropic amblyopia and 111 patients (133 eyes) without amblyopia after lens surgery. The training and validation cohorts were comparable in terms of baseline characteristics (*p* > 0.05).

**Table 1:** Baseline characteristics of the training cohort and validation cohort.

Training cohort (*n* = 496)				Validation cohort (*n* = 209)		
	Amblyopia group	Non-amblyopia group	*p*- value	Amblyopia group	Non-amblyopia group	*p-* value
Subjects/eyes	148/203	195/293		67/76	111/133	
Sex (female: male)	107/96	162 /131	0.648	48/28	73/60	0.308
Eyes (right: left)	108/95	145 /148	0.523	39/37	67/66	0.505
Median age(years)	6 (5 to12)	8 (6 to22)	*<*0.05*	6 (5 to20)	8 (5 to15)	<0.05*
Adult (Age > 15y, %)	48 (23.6%)	90 (30.7%)	0.103	20 (26.3%)	32 (24.1%)	0.741
Cardiac phenotype (%)			*<*0.05**			0.341
Normal (%)	28 (58.33%)	26 (34.2%)		7 (35%)	17 (41.5%)	
Regurgitation (%)	3 (6.25%)	22 (28.9%)		4 (20%)	11 (26.8%)	
Organic (%)	17 (35.42%)	28 (36.8%)		9 (45%)	13 (31.7%)	
Mutation gene			0.195			0.526
*FBN1*	126 (90.7%)	176 (95.2%)		45 (93.7%)	78 (94%)	
*ADAMTSL4*	7 (5%)	4 (2.2%)		2 (4.2%)	1 (1.2%)	
*ADAMTS17*	2 (1.4%)	1 (0.5%)		0 (0%)	2 (2.4%)	
*ASPH*	0 (0%)	3 (1.6%)		0 (0%)	1 (1.2%)	
*CBS*	1 (0.7%)	0 (0%)		1 (2.1%)	1 (1.2%)	
*CPAMD8*	1 (0.7%)	1 (0.5%)		0 (0%)	0 (0%)	
*SUOX*	2 (1.4%)	0 (0%)		0 (0%)	0 (0%)	
Aortic dissection/aortic aneurysm (%)	0 (0%)	3 (10.3%)	0.483	1 (10%)	0 (0%)	0.455
Preoperative parameter						
Preoperative AL (mm)	24.20 (22.57 to 25.76)	23.75 (22.56 to 25.89)	0.666	23.99 (22.26 to 25.57)	23.52 (22.67 to 25.14)	0.629
Z-Preoperative AL	1.85 (−0.18 to 3.82)	0.62 (−0.55 to 2.61)	*<*0.05**	1.38 (−0.21 to 3.57)	0.81 (−0.71 to 2.62)	0.096
Preoperative K1(D)	39.72 (38.76 to 41.09)	40.1 (39.05 to 41.52)	0.053	39.89 (38.41 to 41.23)	39.81 (38.82 to 41.41)	0.428
Preoperative K1 axis	78 (13 to 170)	56 (12 to 163)	0.323	89.5 (13 to 158.75)	128 (12 to 170.5)	0.280
Preoperative K2(D)	41.42 (40.40 to 43.13)	41.88 (40.54 to 43.18)	0.234	41.42 (40.10 to 42.63)	41.8 (40.74 to 43.21)	0.061
Preoperative K2 axis	91 (81 to 104)	91 (78 to 104)	0.776	91.50 (69.75 to 104.50)	88 (78.5 to 100)	0.730
Km (D)	40.51 (39.66 to 41.88)	40.86 (39.76 to 42.31)	0.092	40.62 (39.45 to 41.93)	40.75 (39.85 to 42.30)	0.175
Preoperative ACD (mm)	3.22 (2.85 to 3.57)	3.23 (2.95 to 3.48)	0.833	3.27 (2.89 to 3.65)	3.32 (3.04 to 3.48)	0.743
Preoperative WTW (mm)	12.10 (11.70 to 12.50)	12.1 (11.7 to 12.5)	0.794	12.00 (11.50 to 12.45)	12.1 (11.8 to 12.4)	0.308
Z-Preoperative WTW	0.26 (−0.79 to 1.32)	0.26 (−0.79 to 1.32)	0.909	0.00 (−1.32 to 1.25)	0.26 (−0.53 to 1.05)	0.354
Preoperative BCVA (LogMAR)	0.82 (0.52 to 1.30)	0.40 (0.30 to 0.70)	*<*0.05***	0.70 (0.52 to 1.00)	0.40 (0.30 to 0.70)	*<*0.05***

AL, axial length; K1, flattest keratometry; K2, steepest keratometry; Km, mean keratometry; ACD, anterior chamber depth; WTW, horizontal corneal diameter; BCVA = best-corrected visual acuity. *p < 0.05; **p < 0.01; ***p < 0.001.

### Prevalence of postoperative ametropic amblyopia

**Figure 2:**
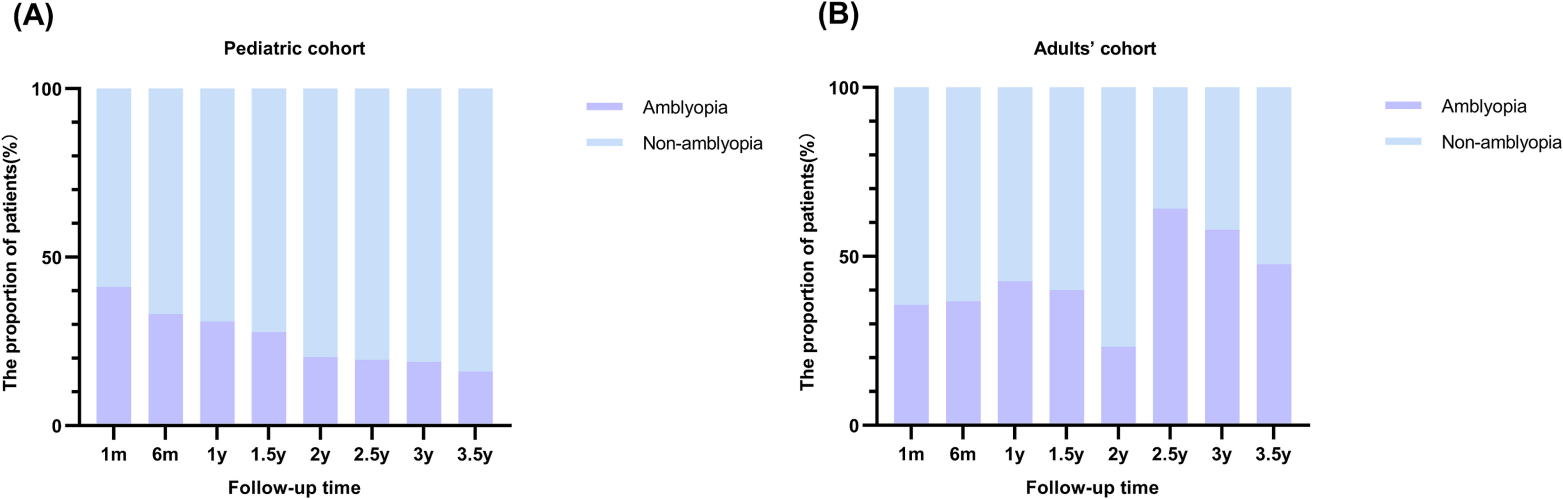
Prevalence of postoperative ametropic amblyopia at various follow-up intervals in pediatric and adults’ cohort. (A) Prevalence of postoperative amblyopia in pediatric cohort. The stacked column chart of the proportion of patients with amblyopia by follow-up time demonstrated a decreasing tendency of amblyopia with longer follow-up time in the pediatric cohort. (B) Prevalence of postoperative amblyopia in adults’ cohort.

As shown in Figure 2, with a follow-up maximum of 3.5 years, the prevalence of amblyopia at various follow-up intervals post-surgery was investigated. Among the pediatric cohort (Figure 2A), there was a noticeable decline in the proportion of the amblyopia population. At 1 month after surgery, the prevalence of amblyopia was 41.13%, which decreased to 16% at 3.5 years after surgery. At 6 months, 1 year, 1.5 years, 2 years, 2.5 years, and 3 years, the prevalence was 33.09, 30.86, 27.67, 20.38, 19.51, and 18.95%, respectively. As for the adults’ cohort, no consistent pattern was identified (Figure 2B).

### Model development

As shown in Table 1, comparative analysis suggested that there were significant differences in age (*p* = 0.029), z-AL (*p* = 0.008), BCVA (*p* < 0.001), and cardiac phenotype (*p* = 0.003) between the amblyopia and non-amblyopia groups. Upon thorough evaluation, a prediction model for postoperative amblyopia was constructed using preoperative biometrics. As shown in Table 2, BCVA and cardiac phenotype were selected as the indexes in the prediction model by multivariate logistic regression. The OR of BCVA was 0.015 (95% CI, 0.002–0.131). Compared with the normal phenotype, the OR of the regurgitation phenotype was 0.06 (95% CI, 0.01–0.345), and the OR of the organic phenotype was 0.235 (95% CI, 0.072–0.764).

**Table 2:** Univariate and multivariate logistic regression models.

	Univariate analysis	*p-* value	Multivariate analysis	*p*- value
	OR (95% CI)		OR (95% CI)	
Z-Preoperative AL	1.094 (1.017–1.017)	0.016	NA	
Cardiac phenotype		0.006		0.003
Cardiac phenotype (1)	0.108 (0.027–0.429)	0.002	0.06 (0.01–0.345)	0.002
Cardiac phenotype (2)	0.479 (0.194–1.186)	0.112	0.235 (0.072–0.764)	0.016
Preoperative BCVA	0.069 (0.037–0.129)	<0.001	0.015 (0.002–0.131)	<0.001

AL, axial length; BCVA, best-corrected visual acuity; NA, not applicable.

### Nomogram construction

**Figure 3:**
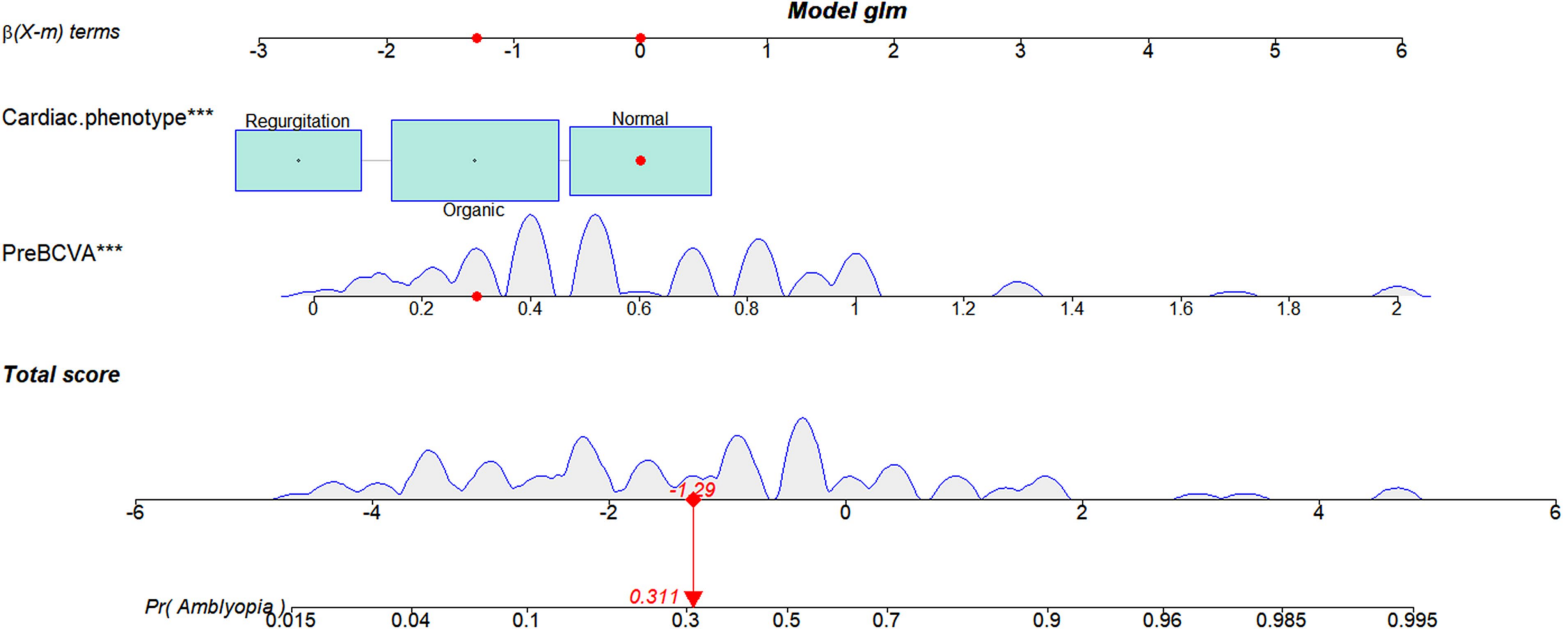
A constructed nomogram for the postoperative amblyopia prediction in a patient with CEL. The importance of each variable was ranked according to the standard deviation along the nomogram scales. For categorical variables, their distributions are reflected by the size of the box (to view boxes of cardiac phenotype, the biggest one represents organic). To use the nomogram, the specific points of individual patients are located on each variable axis. The sum of these points is located on the total score axis, and a line is drawn downward to the amblyopia axes to determine the probability of postoperative amblyopia. preBCVA, preoperative best-corrected visual acuity.

A nomogram was established based on the variables screened to predict the postoperative amblyopia of CEL patients. Figure 3 shows an example of using the nomogram to predict amblyopia in a given patient. For a patient with a normal cardiac phenotype and BCVA of 0.3, the patient’s total score is −1.29, and the probability of amblyopia is 31.1%. Most patients in the present study had total risk points ranging from-5 to 2. If a patient’s total score is 0, the probability of postoperative amblyopia is more than 60%; therefore, more attention should be paid to its subsequent development and follow-up.

### Model performance

**Figure 4:**
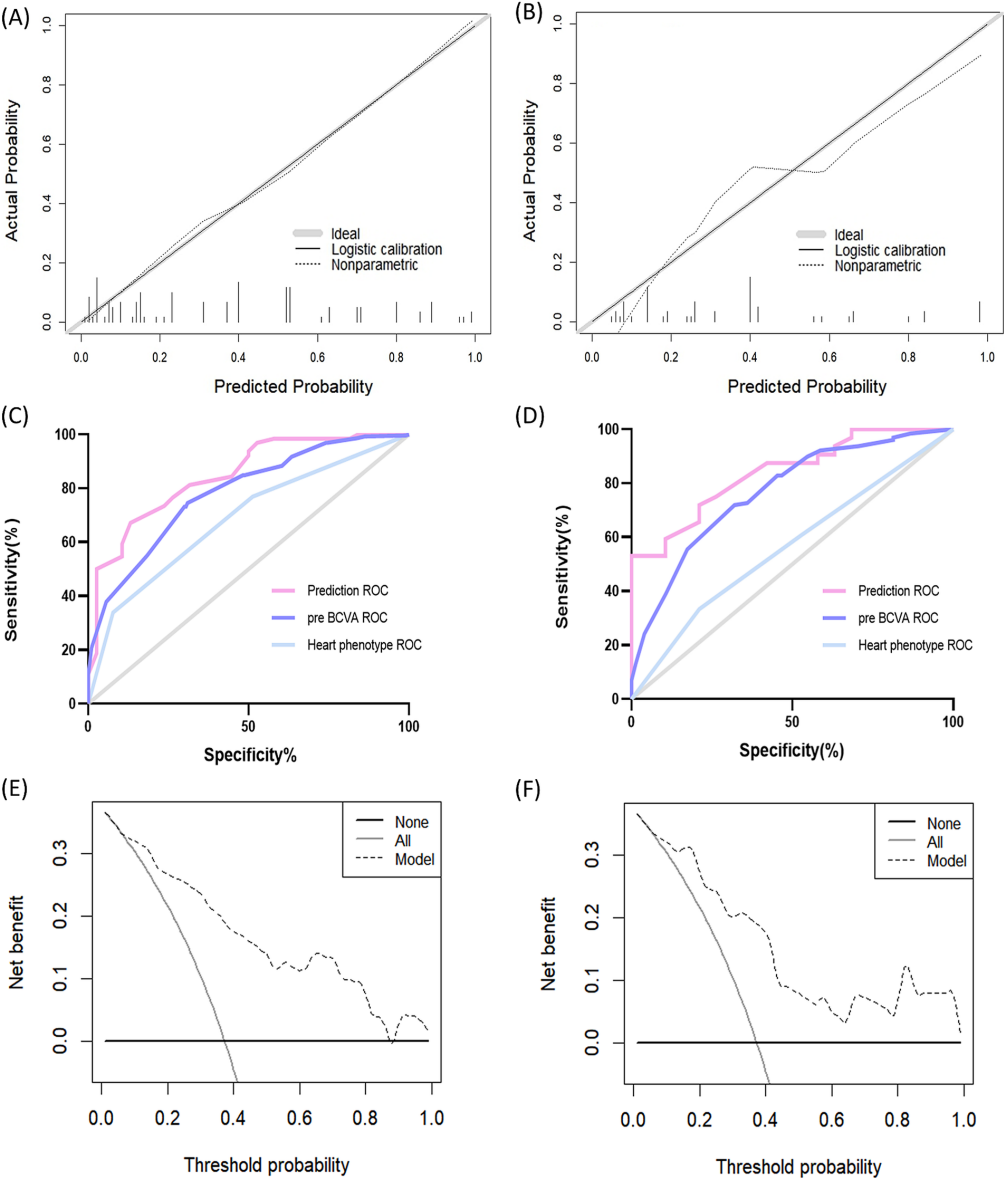
Performance of the prediction model. (A) Calibration curve of the training cohort. The solid curve represents the relationship between the predicted and observed probabilities of postoperative amblyopia. The ideal calibration is represented by the solid curve that fits the gray line exactly. (B) Calibration curve of the validation cohort. (C) ROC curve of the training cohort. The AUC of the prediction model is 0.846 (95% CI, 0.770–0.922). (D) ROC curve of the validation cohort. The AUC of the prediction model in the validation cohort is 0.840 (95% CI, 0.735–0.946). (E) Decision curve analysis of the training cohort. The x-axis determines the threshold probability. The y-axis determines the net benefit. The dotted line represents the amblyopia prediction nomogram.The gray line represents the assumption that all patients have postoperative amblyopia. The black line represents the assumption that all patients do not have postoperative amblyopia. (F) Decision curve analysis of the validation cohort. ROC, Receiver Operating Characteristic; pre BCVA, preoperative best-corrected visual acuity.

The calibration plots of the model for the training and validation cohorts were well-calibrated (Figures 4A,B). The Hosmer-Lemeshow statistical test of the model in the training cohort supported the goodness-of-fit of the model (*χ*^2^ = 9.359; *p* = 0.405). The AUC of the model was 0.846 (95% CI, 0.770–0.922), which was better than each of the two predictors alone, including preoperative BCVA (AUC = 0.731; 95% CI, 0.692–0.786) and cardiac phenotype (AUC = 0.660; 95% CI, 0.565–0.756; Figure 4C). These results indicate favorable discrimination by the prediction model. For the validation cohort, the AUC of the new model was 0.840 (95% CI, 0.735–0.946; Figure 4D). The Hosmer-Lemeshow analysis of the new model in the validation cohort also supported the improved goodness-of-fit of the model (*χ*^2^ = 7.052; *p* = 0.632). The DCA curves used to evaluate the clinical benefit of the nomogram are shown in Figures 4E,F, indicating a good clinical utility of the model.

## Discussion

Pediatric patients with CEL are frequently at risk of postoperative ametropic amblyopia (8–10). Wang et al. have pointed out that amblyopia is one of the most important causes of reduced vision after surgery (10). Beyond the visual deficits associated with amblyopia, there can also be significant psychosocial implications. The Academy of Ophthalmology Preferred Practice Pattern for amblyopia suggests that amblyopia treatment should be considered for children up to the age of 10 years (21), while other studies have indicated that treatment efficacy diminishes beyond the age of 7 years (22–24). Despite this, amblyopia treatment is undoubtedly most effective during the sensitive period in human visual development (15). Delays in the treatment caused by unrecognized conditions can lead to irreversible visual impairment (16). However, in patients with CEL, the lack of consistent predictive markers for postoperative ametropic amblyopia and the intricate interplay of factors such as age, sex, and preoperative conditions make it challenging to identify at-risk patients. Consequently, conducting a large population-based study is imperative to better comprehend the onset and progression patterns of postoperative amblyopia in patients with CEL.

In this study, a large cohort study including 677 patients (889 eyes) operated for CEL was established. Initially, the prevalence of postoperative ametropic amblyopia at various follow-up time points after surgery was investigated. In the pediatric cohort, with the follow-up time from 1 month to 3.5 years, the prevalence of amblyopia showed a notable decreasing trend. Based on our previous research (8), we have advocated for initiating amblyopia therapy 1 month after surgery in clinical practice. This decreasing trend underscores the effectiveness of timely intervention in halting or reversing the progression of ametropic amblyopia and achieving improved visual acuity.

Multiple parameters participate in the postoperative amblyopia of CEL patients. By conducting a comparative analysis between the amblyopic group and the non-amblyopic group, we have observed significant differences in preoperative age, Z-AL, BCVA, and cardiac phenotype in both the training and validation cohorts. Chen et al. reported severe amblyopia is more common in EL children with possible inherent severe phenotypes than in individuals who developed EL in adulthood (8). Consistently, our findings indicate a higher risk of postoperative amblyopia in younger patients, as children are in a critical period of visual development. In terms of ocular parameters, poorer BCVA correlates with worse visual outcomes. This observation is supported by a previous study that good vision prior to the onset of visually significant lens subluxation generally predicts a more favorable prognosis (6). Additionally, a study about the impact of preoperative AL on the myopic shift postoperatively suggested that AL may significantly influence the refractive outcomes after lens surgery (25). Similarly, in our study, we have also observed that longer AL is a risk factor for postoperative amblyopia in CEL patients.

Since CEL is often associated with inherited connective tissue disorders like Marfan syndrome, which involves aortic root dilation, mitral valve prolapse, and ectopia lentis, we also considered the cardiac phenotype as a potential risk factor for postoperative amblyopia (26, 27). Surprisingly, a normal cardiac phenotype was linked to worse visual outcomes and a higher risk of amblyopia compared to regurgitation or organic phenotypes. Although the correlations between ocular and cardiovascular phenotypes in children with CEL have been reported (28), this is the first study to link cardiac phenotype with the visual prognosis of CEL patients. Consequently, the pathogenesis behind the increased amblyopia risk remains unclear. However, this novel finding highlights the need for further research to understand the underlying mechanisms and the importance of monitoring cardiac manifestations in these patients.

Thus, we propose that preoperative assessment of age, BCVA, Z-AL combined with cardiac phenotype has the potential to be sensitive and specific predictors for the occurrence of postoperative ametropic amblyopia. A prediction model and a nomogram were created to assess patients’ probability of postoperative amblyopia. Several advantages of our study that should be considered. First of all, the development of the prediction model for postoperative amblyopia enables clinicians to identify high-risk patients early, allowing for timely interventions to mitigate visual impairment. By integrating preoperative ocular metrics and patient-specific risk factors, the model provides a personalized approach to postoperative care. Moreover, it contributes to the optimization of treatment strategies, enhancing visual outcomes and reducing the long-term burden of postoperative amblyopia. Furthermore, with a cohort of 889 eyes from 677 CEL patients, our study is the first and largest known cohort study to comprehensively reveal the onset and progression patterns of postoperative amblyopia in patients with CEL, and it demonstrated the effectiveness of timely amblyopia treatment in a pediatric cohort.

We acknowledge some limitations of this study that warrant consideration. First of all, the patients were enrolled from a single ophthalmic center, and external validation might be necessary in the future. Additionally, due to long-term follow-up, some ocular characteristics were missing; therefore, we excluded patients missing some important examination details. Moreover, as this is the first study to explore the relationship between cardiac phenotype and visual prognosis in CEL patients, further longitudinal studies with larger sample sizes are required to confirm these findings.

In conclusion, a decreasing trend in the prevalence of ametropic amblyopia was investigated in pediatric CEL patients. A new model and a nomogram were built for amblyopia prediction, which showed good performance in both the training cohort and the validation cohort. We hope this prediction model will facilitate precise and convenient application, thereby guiding clinicians in optimizing early diagnostic strategies and timely treatment for patients with postoperative amblyopia.

## Data Availability

The original contributions presented in the study are included in the article/supplementary material, further inquiries can be directed to the corresponding author/s.
