# Corrigendum: Clinical ocular prediction model of postoperative ametropic amblyopia in patients with congenital ectopia lentis

**DOI:** 10.3389/fmed.2024.1532287

**Published:** 2024-12-12

**Authors:** Xinyue Wang, Linghao Song, Yan Liu, Qiuyi Huo, Yang Sun, Zexu Chen, Wannan Jia, Xin Shen, Yalei Wang, Xinyao Chen, Tianhui Chen, Yongxiang Jiang, Rui Wang

**Affiliations:** ^1^Department of Ophthalmology, The First Affiliated Hospital of Northwest University, Xi'an, China; ^2^Eye Institute and Department of Ophthalmology, Eye & ENT Hospital, Fudan University, Shanghai, China; ^3^Key Laboratory of Myopia and Related Eye Diseases, NHC; Key Laboratory of Myopia and Related Eye Diseases, Chinese Academy of Medical Sciences, Shanghai, China; ^4^Shanghai Key Laboratory of Visual Impairment and Restoration, Shanghai, China

**Keywords:** congenital ectopia lentis, ametropic amblyopia, nomogram, prediction model, visual prognosis

In the published article, there was an error in affiliation(s) [1,2,3,4]. Instead of

“[Xinyue Wang^1, 2, 3†^, Linghao Song^1, 2, 3†^, Yan Liu^1, 2, 3^, Qiuyi Huo^1, 2, 3^, Yang Sun^1, 2, 3^, Zexu Chen^1, 2, 3^, Wannan Jia^1, 2, 3^, Xin Shen^1, 2, 3^, Yalei Wang^1, 2, 3^, Xinyao Chen^1, 2, 3^, Tianhui Chen^1, 2, 3*^, Yongxiang Jiang^1, 2, 3*^ and Rui Wang^4*^

^1^Eye Institute and Department of Ophthalmology, Eye and ENT Hospital, Fudan University, Shanghai, China

^2^NHC Key Laboratory of Myopia, Key Laboratory of Myopia, Fudan University, Chinese Academy of Medical Sciences, Shanghai, China

^3^Shanghai Key Laboratory of Visual Impairment and Restoration, Shanghai, China

^4^Department of Ophthalmology, The First Affiliated Hospital of Northwest University, Xi'an, China]

“It should be”

[Xinyue Wang^2, 3, 4†^, Linghao Song^2, 3, 4†^, Yan Liu^2, 3, 4^, Qiuyi Huo^2, 3, 4^, Yang Sun^2, 3, 4^, Zexu Chen^2, 3, 4^, Wannan Jia^2, 3, 4^, Xin Shen^2, 3, 4^, Yalei Wang^2, 3, 4^, Xinyao Chen^2, 3, 4^, Tianhui Chen^2, 3, 4*^, Yongxiang Jiang^2, 3, 4*^ and Rui Wang^1*^

^1^Department of Ophthalmology, The First Affiliated Hospital of Northwest University, Xi'an, China]”.

^2^Eye Institute and Department of Ophthalmology, Eye & ENT Hospital, Fudan University, Shanghai, China

^3^Key Laboratory of Myopia and Related Eye Diseases, NHC; Key laboratory of Myopia and Related Eye Diseases, Chinese Academy of Medical Sciences, Shanghai, China

^4^Shanghai Key Laboratory of Visual Impairment and Restoration, Shanghai, China

The authors apologize for this error and state that this does not change the scientific conclusions of the article in any way. The original article has been updated.

